# Association between oral anticoagulants and COVID-19-related outcomes: a population-based cohort study

**DOI:** 10.3399/BJGP.2021.0689

**Published:** 2022-04-20

**Authors:** Angel YS Wong, Laurie Tomlinson, Jeremy P Brown, William Elson, Alex J Walker, Anna Schultze, Caroline E Morton, David Evans, Peter Inglesby, Brian MacKenna, Krishnan Bhaskaran, Christopher T Rentsch, Emma Powell, Elizabeth Williamson, Richard Croker, Seb Bacon, William Hulme, Chris Bates, Helen J Curtis, Amir Mehrkar, Jonathan Cockburn, Helen I McDonald, Rohini Mathur, Kevin Wing, Harriet Forbes, Rosalind M Eggo, Stephen JW Evans, Liam Smeeth, Ben Goldacre, Ian J Douglas, (The OpenSAFELY Collaborative)

**Affiliations:** Faculty of Epidemiology and Population Health, London School of Hygiene and Tropical Medicine, London.; Faculty of Epidemiology and Population Health, London School of Hygiene and Tropical Medicine, London.; Faculty of Epidemiology and Population Health, London School of Hygiene and Tropical Medicine, London.; Faculty of Epidemiology and Population Health, London School of Hygiene and Tropical Medicine, London.; The DataLab, Nuffield Department of Primary Care Health Sciences, University of Oxford, Oxford.; Faculty of Epidemiology and Population Health, London School of Hygiene and Tropical Medicine, London.; The DataLab, Nuffield Department of Primary Care Health Sciences, University of Oxford, Oxford.; The DataLab, Nuffield Department of Primary Care Health Sciences, University of Oxford, Oxford.; The DataLab, Nuffield Department of Primary Care Health Sciences, University of Oxford, Oxford.; The DataLab, Nuffield Department of Primary Care Health Sciences, University of Oxford, Oxford.; Faculty of Epidemiology and Population Health, London School of Hygiene and Tropical Medicine, London.; Faculty of Epidemiology and Population Health, London School of Hygiene and Tropical Medicine, London.; Faculty of Epidemiology and Population Health, London School of Hygiene and Tropical Medicine, London.; Faculty of Epidemiology and Population Health, London School of Hygiene and Tropical Medicine, London.; The DataLab, Nuffield Department of Primary Care Health Sciences, University of Oxford, Oxford.; The DataLab, Nuffield Department of Primary Care Health Sciences, University of Oxford, Oxford.; The DataLab, Nuffield Department of Primary Care Health Sciences, University of Oxford, Oxford.; TPP, Leeds.; The DataLab, Nuffield Department of Primary Care Health Sciences, University of Oxford, Oxford.; The DataLab, Nuffield Department of Primary Care Health Sciences, University of Oxford, Oxford.; TPP, Leeds.; Faculty of Epidemiology and Population Health, London School of Hygiene and Tropical Medicine, London and NIHR Health Protection Research Unit (HPRU) in Immunisation, London School of Hygiene and Tropical Medicine, London.; Faculty of Epidemiology and Population Health, London School of Hygiene and Tropical Medicine, London.; Faculty of Epidemiology and Population Health, London School of Hygiene and Tropical Medicine, London.; Bristol Medical School, University of Bristol, Bristol.; Faculty of Epidemiology and Population Health, London School of Hygiene and Tropical Medicine, London.; Faculty of Epidemiology and Population Health, London School of Hygiene and Tropical Medicine, London.; Faculty of Epidemiology and Population Health, London School of Hygiene and Tropical Medicine, London and NIHR Health Protection Research Unit (HPRU) in Immunisation, London School of Hygiene and Tropical Medicine, London.; The DataLab, Nuffield Department of Primary Care Health Sciences, University of Oxford, Oxford.; Faculty of Epidemiology and Population Health, London School of Hygiene and Tropical Medicine, London.

**Keywords:** COVID-19, warfarin, dabigatran, Factor Xa Inhibitors

## Abstract

**Background:**

Early evidence has shown that anticoagulant reduces the risk of thrombotic events in those infected with COVID-19. However, evidence of the role of routinely prescribed oral anticoagulants (OACs) in COVID-19 outcomes is limited.

**Aim:**

To investigate the association between OACs and COVID-19 outcomes in those with atrial fibrillation and a CHA_2_DS_2_-VASc score of 2.

**Design and setting:**

On behalf of NHS England, a population-based cohort study was conducted.

**Method:**

The study used primary care data and pseudonymously-linked SARS-CoV-2 antigen testing data, hospital admissions, and death records from England. Cox regression was used to estimate hazard ratios (HRs) for COVID-19 outcomes comparing people with current OAC use versus non-use, accounting for age, sex, comorbidities, other medications, deprivation, and general practice.

**Results:**

Of 71 103 people with atrial fibrillation and a CHA_2_DS_2_-VASc score of 2, there were 52 832 current OAC users and 18 271 non-users. No difference in risk of being tested for SARS-CoV-2 was associated with current use (adjusted HR [aHR] 0.99, 95% confidence interval [CI] = 0.95 to 1.04) versus non-use. A lower risk of testing positive for SARS-CoV-2 (aHR 0.77, 95% CI = 0.63 to 0.95) and a marginally lower risk of COVID-19-related death (aHR, 0.74, 95% CI = 0.53 to 1.04) were associated with current use versus non-use.

**Conclusion:**

Among those at low baseline stroke risk, people receiving OACs had a lower risk of testing positive for SARS-CoV-2 and severe COVID-19 outcomes than non-users; this might be explained by a causal effect of OACs in preventing severe COVID-19 outcomes or unmeasured confounding, including more cautious behaviours leading to reduced infection risk.

## INTRODUCTION

Early studies have reported that heparin lowers the risk of pulmonary embolism and mortality during hospital admissions in patients with COVID-19.^1^^–^4 However, the protective role of oral anticoagulants (OACs) in preventing severe COVID-19 outcomes is unclear. A randomised controlled trial has reported that therapeutic anticoagulation with rivaroxaban in patients admitted to hospital with COVID-19 did not improve all-cause mortality but increased the risk of major bleeding compared with prophylactic anticoagulation with enoxaparin or unfractionated heparin during hospital admissions.^5^ Notably, as this study excluded people with an indication for therapeutic anticoagulation, it remains uncertain whether there is an effect of routinely prescribed OACs for atrial fibrillation on COVID-19 outcomes. In particular, anticoagulants are underutilised among people with atrial fibrillation in the UK.^6^ Understanding the effects of routinely prescribed OACs on COVID-19 may be of significant clinical importance that further informs OAC prescribing guidance in the context of the COVID-19 pandemic, given that people with atrial fibrillation are at higher risk of severe COVID-19 outcomes.^7^

Some observational studies have investigated the role of OACs in COVID-19 outcomes but the findings were conflicting.^8^^–^19 Importantly, most studied were of small sample size or in hospital settings only, leading to a lack of power and/or selection bias. In addition, people taking OACs are likely to have more comorbidities than those who are not, and so comparing COVID-19 outcomes for OAC use with non-use could be subject to confounding in these studies.

In order to address these limitations, a population-based cohort study was conducted to investigate the association between routinely prescribed OACs and COVID-19 outcomes versus non-use, restricting the study population to people in England with atrial fibrillation who had a CHA_2_DS_2_-VASc score of 2. The CHA_2_DS_2_-VASc score is a validated measure to predict the risk of stroke among people with atrial fibrillation. The use of the CHA_2_DS_2_-VASc score is to determine the need for prescribed anticoagulants in people with non-valvular atrial fibrillation as a prophylactic therapy against stroke. According to the guidelines for the management of patients with atrial fibrillation,^20^^–^22 people with a CHA_2_DS_2_-VASc score of ≥2 should be offered an anticoagulant. For those with a score of 2, there is possibly a degree of variation in OAC prescribing, offering a useful group in which OAC users are likely to be more comparable with non-users to minimise confounding. Also, a UK study has shown that patients with a CHA_2_DS_2_-VASc score of 2 were more likely to remain untreated with anticoagulants than patients with a score of ≥3.^6^ A better understanding of the impact of OACs on COVID outcomes may alter the balance of benefits and risks of prescribing OACs for those around such a threshold.

**Table:** How this fits in

Early studies reported that heparin lowers the risk of pulmonary embolism and mortality during hospitalisation among patients with COVID-19. However, the protective role of oral anticoagulants in severe COVID-19 outcomes is unclear. This study showed a lower risk of testing positive for SARS-CoV-2 and COVID-19-related deaths associated with current OAC use, versus non-use among people with atrial fibrillation and a low baseline risk of stroke.

## METHOD

### Study design

A population-based cohort study was conducted between 1 March 2020 and 28 September 2020.

### Data source

Primary care records managed by the software provider TPP were linked to SARS-CoV-2 antigen testing data from the Second Generation Surveillance System, COVID-19-related hospital admissions from the Secondary Uses Service, and Office for National Statistics death data through OpenSAFELY, a data analytics platform created by the author team on behalf of NHS England.^23^ The dataset analysed within OpenSAFELY is based on 24 million people currently registered with primary care practices using TPP SystmOne software, representing 40% of the English population. It includes pseudonymised data such as coded diagnoses, prescribed medications, and physiological parameters. Details on information governance can be found in Supplementary Information S1.

### Study populations

People with a diagnosis of atrial fibrillation on or before the study start date (1 March 2020) were identified. To reduce confounding, the cohort was limited to those with a CHA_2_DS_2_-VASc score of 2, as the indication for OAC therapy would typically be borderline among people with this score. Their CHA_2_DS_2_-VASc score was calculated based on their records relating to demographics and diagnoses, which contributed to the score before cohort entry.

People were excluded if they had missing data for sex, Index of Multiple Deprivation, <1 year of primary care records, or aged <18 years or >110 years and prescribed injectable anticoagulants 4 months before the study start date.

### Exposure

Current OAC users were people who ever had an OAC prescription (that is warfarin, dabigatran, rivaroxaban, apixaban, edoxaban) in the 4 months before the study start, and the non-users were people with no record of an OAC prescription in the same period.

### Outcomes and follow-up

The outcomes were:

- testing positive for SARS-CoV-2;
- a COVID-19-related hospital admission; and
- a COVID-19-related death (defined as the presence of ICD-10 codes U071 [confirmed COVID-19] and U072 [suspected COVID-19] on the death certificate).

Testing outcomes were obtained from the UK’s Pillar 1 (NHS and Public Health England laboratories) and Pillar 2 (commercial partners) testing strategies and results from polymerase chain reaction (PCR) swab tests used to identify individuals who were symptomatic.^24^ As pre-specified analyses, time to the first SARS-CoV-2 test was also included as a negative control outcome. It was anticipated that, within this population of people with atrial fibrillation, there were unlikely to be marked differences in the likelihood of being tested for SARS-CoV-2 infection in relation to drug treatment with OACs.

**Figure 1.**
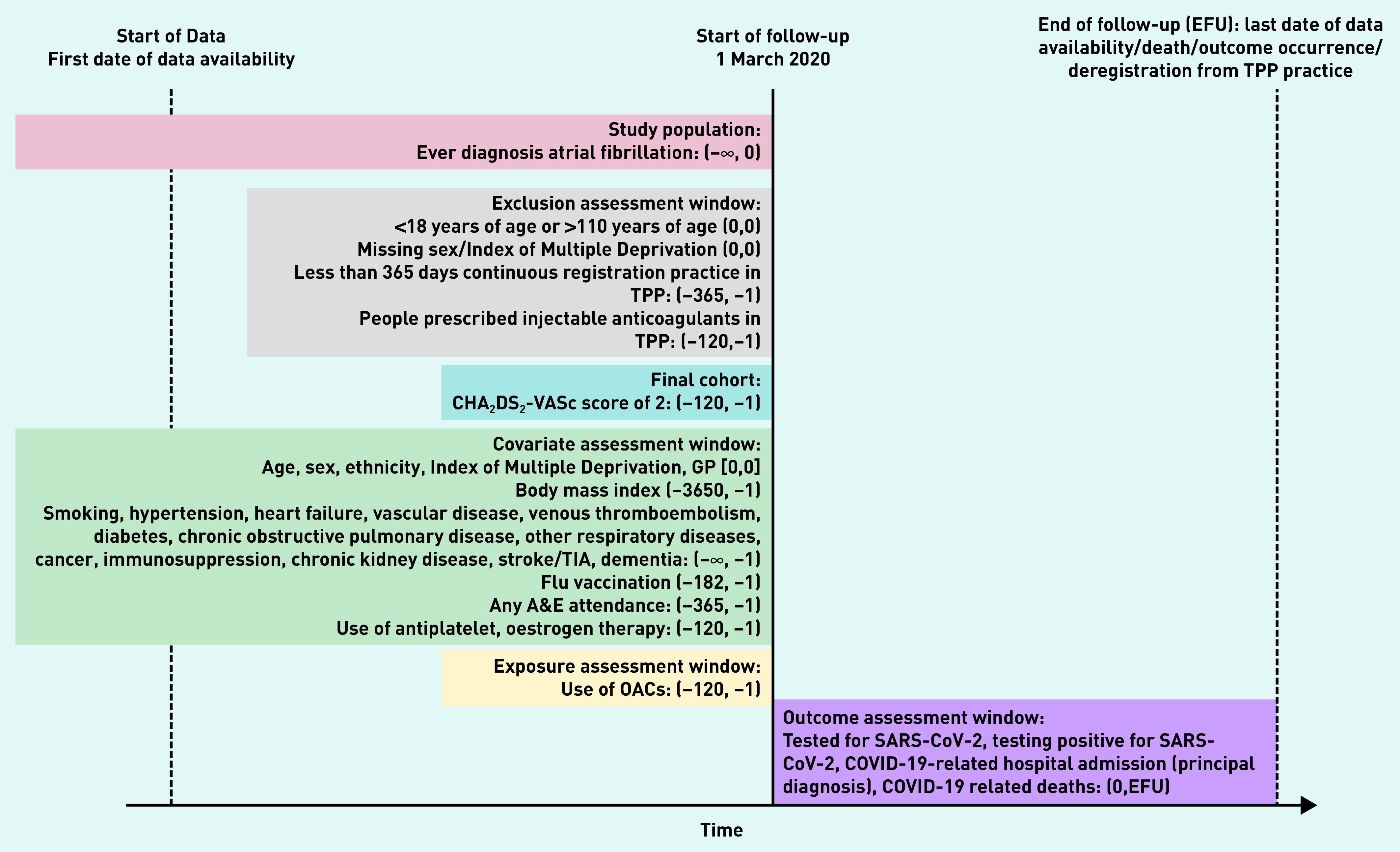
*Study diagram, illustrating the follow-up from 1 March 2020 to the latest at death, outcome occurrence, deregistration from TPP (TPP SystmOne software practice), or 28 September 2020 and identification of covariates before 1 March 2020. Date in brackets shows the range of the time window (days in unit). A&E = accident & emergency. EFU = end of followup. OAC = oral coagulation. TIA = transient ischaemic attack.*

Follow-up began on the 1 March 2020 and ended at the latest at the outcome of interest in each analysis, deregistration from the TPP practice, death, or study end date (28 September 2020) (Figure 1).

### Covariates

Clinical covariates were identified using the Read clinical classification system and included body mass index (BMI) to classify obesity and smoking status. Obesity was classified using BMI in kg/m^2^: no record of obesity or BMI <30.0; obese I (BMI 30.0–34.9 kg/m^2^); obese II (BMI 35.0–39.9 kg/m^2^) and obese III (≥40 kg/m^2^). Smoking status was grouped into current, former, and never smokers. Those with missing smoking status were categorised as never smokers. The following covariates were pre-specified using a directed acyclic graph (DAG) (Supplementary Figure S1), including age, sex, obesity, smoking status, hypertension, heart failure, myocardial infarction, peripheral arterial disease, stroke/transient ischaemic attack, venous thromboembolism, diabetes, flu vaccination, current antiplatelet use, current oestrogen/oestrogen-like therapy use, and Index of Multiple Deprivation. Dementia was added to the regression during the peer-review process as it could be an important confounder. The authors of the present study identified these covariates, which are both associated with the exposure to OACs and severe COVID-19 outcomes.^23^ Some covariates that are associated with the exposure and venous thromboembolism, possibly leading to severe COVID-19 outcomes, were also included.^25^^,^^26^ All code lists for identifying exposures, covariates, and outcomes are openly shared at https://codelists.opensafely.org/.

### Statistical methods

Adjusted cumulative incidence/mortality curves are presented using the Royston-Parmar model. Hazard ratios (HRs) with 95% confidence intervals (CIs) were estimated using Cox regression with time since cohort entry as the underlying timescale. Competing risk was accounted for by modelling the cause-specific hazard (that is censoring non-COVID-19 other deaths for COVID-19 death analysis, and censoring any death for other outcomes analysis). Graphical methods and tests based on Schoenfeld residuals were used to explore violations of the proportional hazards assumption.

Unadjusted models, models adjusted for age (using restricted cubic splines) and sex, and a DAG-adjusted model were constructed.

### Quantitative bias analysis

The authors considered that OAC users may lower their COVID-19 risk through more risk-averse health behaviour (for example wearing face masks, avoiding close proximity to others) than non-users. Given that health behaviour is not captured in databases, quantitative bias analyses (both the E-value and the Cornfield condition)^27^ were conducted to assess the sensitivity of the results to this potential unmeasured confounder.

### Sensitivity analyses

Box 1 shows the list of sensitivity analyses.

**Box 1.** List of sensitivity analyses

**Sensitivity analysis**	**Justification**
1. In addition to the covariates identified by DAG, other covariates based on prior evidence of likely confounders such as chronic obstructive pulmonary disease, other respiratory diseases, cancer, immunosuppression, chronic kidney disease, general practice attendance rate in the year before cohort entry, and A&E attendance rate in the year before cohort entry were also included in the fully adjusted models (stratified by general practice)	To test the robustness of the covariate selection
2. Additionally adjusted for ethnicity in DAG and fully adjusted models. In the fully adjusted models, additional covariates included chronic obstructive pulmonary disease, other respiratory diseases (not including asthma), cancer, immunosuppression, chronic kidney disease, general practice attendance rate in the year before cohort entry, and A&E attendance rate in the year before cohort entry	In the main analysis, ethnicity is not adjusted for because of a sizable proportion of individuals with missing ethnicity (∼23%). Complete case analysis was undertaken to address missing data
3. Repeated main analysis excluding people prescribed antiplatelets 4 months before study start date	To explore the impact of use of antiplatelets, which can reduce the risk of blood clots
4. Limited the study cohort to people aged ≥55 years	To explore the impact of potential confounders at a young age, for example, pregnancy
5. Stratified the cohort by care home residence for the outcome of testing positive for SARS-CoV-2 (*post hoc* analysis)	To explore the impact of health behaviour, as people living in care homes were less likely to have differences in behaviour

*A&E = accident & emergency. DAG = directed acyclic graph.*

Data management was performed using Python 3.8 and SQL, with analysis carried out using Stata 16.1. All study analyses were pre-planned unless otherwise stated. All code for data management and analyses are archived at https://github.com/opensafely/oral-anticoagulant-covid outcome, in addition to the pre-specified protocol (https://github.com/opensafely/anticoagulants-research/blob/master/protocol/Protocol_%20Anticoag%20OpenSAFELY_v3.docx). Deviations from pre-specified protocol, with reasons are provided in Supplementary Information S2 and Supplementary Table S1.

## RESULTS

**Figure 2.**
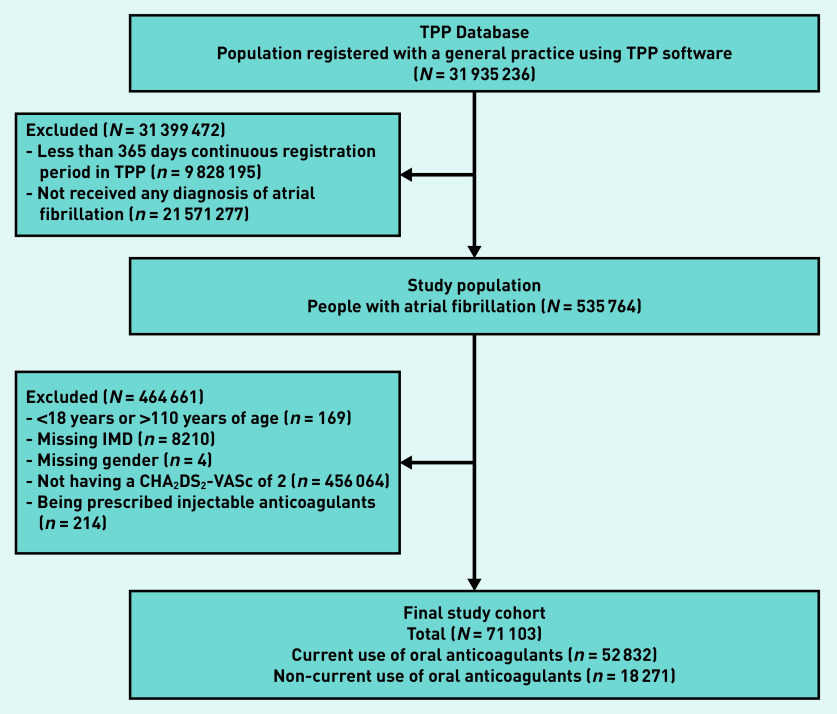
*Flow chart of inclusion of participants. IMD = Index of Multiple Deprivation. TPP = TPP SystmOne software practice.*

Of 71 103 people with atrial fibrillation and a CHA_2_DS_2_-VASc score of 2, there were 52 832 current OAC users and 18 271 non-users (Figure 2, Supplementary Table S2). The median age was 71 years (interquartile range [IQR] 66–75 years) among current users and 69 years (IQR 63–74 years) among non-users.

Current OAC users were more likely to be male, obese, former smokers, and have a medical history of heart failure, chronic obstructive pulmonary disease, and chronic kidney disease, but were less likely to have myocardial infarction, peripheral artery disease, venous thromboembolism, immunosuppression, and diabetes than non-users. Current users were less likely to have a prescription for oestrogen/oestrogen-like drugs, antiplatelets, non-steroidal anti-inflammatory drugs, and aspirin, but to have had more primary care consultations and a flu vaccination than non-users.

**Figure 3.**
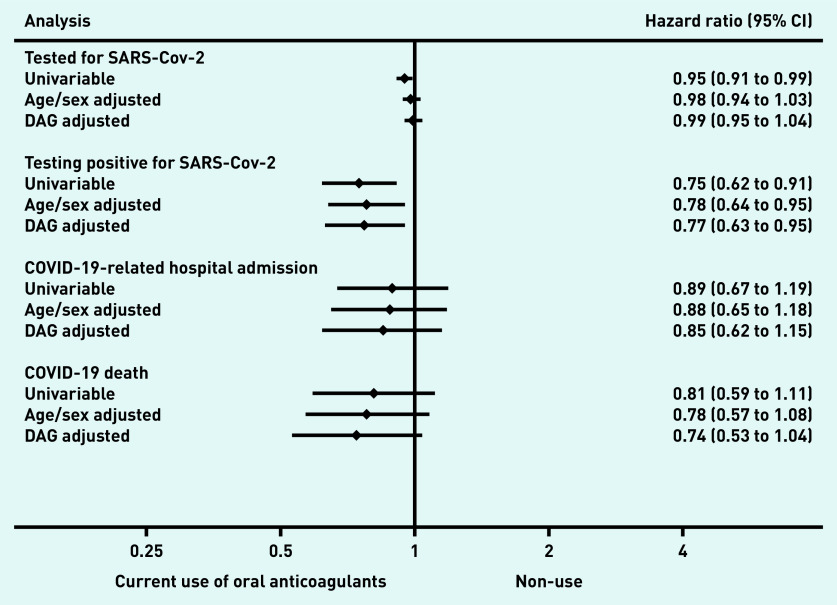
*Hazard ratios of the association between current use of oral anticoagulants and COVID-19-related outcomes versus non-use in people with atrial fibrillation who had a CHA2DS2-VASc score of 2. CI = confidence interval. DAG = directed acyclic graph.*

Supplementary Figure S2 presents time to COVID-19-related outcomes in adjusted cumulative incidence plots. A lower risk of testing positive for SARS-CoV-2 (480 events in 2 107 517 person-weeks; unadjusted HR 0.75 [95% CI = 0.62 to 0.91]; DAG-adjusted HR 0.77 [95% CI = 0.63 to 0.95]) was associated with current use, compared with non-use (Figure 3, Supplementary Table S3). Similarly, a lower risk of COVID-19-related hospital admission was observed with wide CIs (226 events in 2 110 854 person-weeks; unadjusted HR 0.89 [95% CI = 0.67 to 1.19]; DAG-adjusted HR 0.85 [95% CI:0.62 to 1.15]), and COVID-19-related deaths (185 events in 2 113 796 person-weeks; unadjusted HR 0.81 [95% CI = 0.59 to 1.11]; DAG-adjusted HR 0.74 [95% CI = 0.53 to 1.04]), comparing current use with non-use.

For the negative control outcome, there was no difference in risk of being tested for SARS-CoV-2 comparing current use with non-use (11 190 events in 1 994 072 person-weeks; unadjusted HR 0.95 [95% CI = 0.91 to 0.99]; DAG-adjusted HR 0.99 [95% CI = 0.95 to 1.04]) (see Figure 3 & Supplementary Table S3).

### Sensitivity analyses

In the fully adjusted model, a lower risk of COVID-19-related death comparing current OAC use with non-use (HR 0.66, 95% CI = 0.45 to 0.97) was observed. Results of all other sensitivity analyses were similar to those in the main analyses (Supplementary Tables S4–S6). Although there was no strong evidence of a different association between current use of OACs and testing positive for SARS-CoV-2 according to care home residence (Supplementary Table S7), this comparison was underpowered.

### Quantitative bias analysis

To potentially fully explain the observed associations, either non-anticoagulant use would need to be associated with at least a 1.29 increased risk of unmeasured risk-prone behaviour; or risk-prone behaviour would need to be associated with a 1.29 times increased risk of each COVID-19 outcome. Alternatively, both non-use and each COVID-19 outcome would need to be associated with at least a 1.05 times increased risk or unmeasured risk-prone behaviour (Supplementary Table S8).

## DISCUSSION

### Summary

A lower risk of testing positive for SARS-CoV-2 was associated with current OAC use versus non-use among people with atrial fibrillation and a low baseline risk of stroke. With small absolute numbers of COVID-19-related hospital admissions and COVID-19-related deaths, a marginally lower risk of these outcomes was associated with current OAC use compared with non-use. No difference in the risk of being tested for SARS-CoV-2 between current users and non-users was found, indicating that the lower risk of testing positive was unlikely to be because of the chance of being tested.

Consideration needs to be given to whether these associations are causal, or because of other differences between groups. There is no clear evidence that the current users were generally frailer in terms of their comorbidities than non-users. The inverse associations in OAC users were specific to COVID-19 outcomes, with no protective association seen against having a COVID-19 test, which would support a possible causal association. OAC users had a reduced risk of receiving a positive test and severe COVID-19-related outcomes, suggesting a lower risk of acquiring test-detected infection in this group. An experimental study suggested that direct factor Xa inhibitors may prevent SARS-CoV entry to human cells by preventing the spike protein cleavage into the S1 and S2 subunits^28^ but the clinical evidence is limited. Additionally, the authors of the present study considered that anticoagulation might inhibit the PCR for SARS-CoV-2^29^^,^^30^ but the evidence of a specific inhibitory effect of OACs on PCRs for SARS-CoV-2 is lacking. Notably, these results do not necessarily mean OACs reduce the risk of infection; in many instances the outcome of a positive COVID-19 test reflects both infection and symptom severity leading to test seeking. It is possible that anticoagulant activity lessens symptoms/severity, leading to a lower rate of infection detection.

The authors of the present study have also considered the non-causal explanation that risk behaviours may differ between OAC users and non-users and this may have an impact on the probability of being infected with SARS-CoV-2 and severe COVID-19 outcomes. Although the authors cannot fully capture the behavioural differences between groups in the database, it was observed that OAC users were less likely to be current smokers, had less hazardous alcohol use, and were more likely to have had flu vaccination than non-users but the differences were small. A quantitative bias analyses was performed and found that an unmeasured confounder would need to be of moderate strength to potentially explain the observed associations. Although this is always possible, notably the very wide range of well measured confounders the authors did have information on had little impact on these findings, suggesting confounding may not be a substantial problem. Nonetheless, further studies that can account for behavioural differences between groups are required to confirm the findings as the authors of the present study cannot rule this out as a possible contributor to these findings.

### Strengths and limitations

The greatest strength of this study was the power enabling the examination of the association between OACs and various COVID-19 outcomes as the dataset included medical records from 24 million individuals. A quantitative bias analyses was also conducted to explore the impact of unmeasured confounding on the observed results, complementing the interpretation. The breadth of data available in primary care makes it possible to account for a wide range of potential confounders.

Possible limitations are recognised. First, as in any observational study, residual confounding could not be eliminated. Further, there may be misclassification bias in measuring covariates, leading to incomplete adjustment for confounding. Although the authors of the present study attempted to reduce confounding by limiting the cohort to people who had a threshold CHA_2_DS_2_-VASc score for being prescribed anticoagulants, results may not be generalisable to all patients with atrial fibrillation. In particular, females, people with stroke, transient ischaemic attack or venous thromboembolism may be underrepresented in this study given that these alone would have led to a CHA_2_DS_2_-VASc score of 2, so any additional risk factors would mean therefore they would be excluded from the exposed group.

Second, it is not known whether patients took the medications as prescribed. However, non-adherence of OAC treatment would only bias the estimate towards null.

Third, it was not possible to capture anticoagulant use (for example low molecular weight heparin or unfractionated heparin) during admissions to hospital. However, this would tend to make the comparison groups more similar to each other during admissions to hospital, which would most likely lead to an underestimation of any effect of routine OAC use before hospital admission.

Fourth, there may be misclassification in ascertaining atrial fibrillation using diagnostic codes alone when deriving the study population. Some recorded atrial fibrillation might resolve at the study start and thus not require anticoagulants. However, this is considered to be less common^31^ and is unlikely to substantially bias these results. Fifth, some cases of people having COVID-19 in the early pandemic may have been missed because of limited testing capacity. Importantly, the effect estimate in this analysis would not be biased assuming non-differential misclassification bias of outcome.

### Comparison with existing literature

Although the effects of COVID-19 may predispose patients to thromboembolic disease through severe illness, hypoxia, or severe inflammatory response,^32^ anticoagulation may have a role in preventing thrombotic events in patients with COVID-19. Recent studies investigating the potential effects of early initiation of anticoagulation resulted in conflicting findings.^3^^,^^8^^–^15^,^^33^^–^37

Seven studies^8^^,^^9^^,^^13^^,^^14^^,^^16^^–^18 focusing on prehospital use of anticoagulants and one study^34^ focusing on therapeutic use of anticoagulants found no difference in risk of mortality, mechanical ventilation, or acute respiratory distress syndrome. Notably, some were of small sample size,^8^^,^^16^^–^18 with unclear exposure definition,^9^ classifying patients who initiated therapeutic anticoagulation on day 3 or later after intensive care unit admission in the control group^34^ or used composite outcomes with varying clinical importance,^13^^,^^14^ limiting the interpretation of the results. Two cohort studies showed a higher risk of admission to an intensive care unit, intubation, or death associated with anticoagulants in patients with COVID-19 disease versus non-use without restricting to a study population with a specific OAC indication, but the findings were possibly the result of confounding by indication.^12^^,^^36^ A cohort study with propensity score matching reported a lower risk of all-cause mortality associated with OACs in people with COVID-19 disease compared with non-use, supporting the results in the present study.^19^

### Implications for research and practice

Notably, this study was undertaken during the early pandemic before vaccines and COVID-19 treatments were available to prevent or treat severe COVID-19 disease. Future work is needed to confirm these findings of an inverse association between OACs and severe COVID-19-related outcomes in people with atrial fibrillation at the threshold for OAC treatment, and to establish causality; either randomised trials or observational studies with detailed data on risk factors for COVID-19 infection. If confirmed to be a causal effect, this could be of significant clinical importance, particularly as the older age and comorbidities in this group are independent risk factors for severe COVID-19 outcomes. Choice of whether to prescribe routine anticoagulant therapy represents a complex balance of expected risks, benefits, and patient preference; the authors do not recommend changes to ongoing anticoagulant therapy based on these results.

## Data

All data were linked, stored, and analysed securely within the OpenSAFELY platform. Detailed pseudonymised patient data are potentially re-identifiable and therefore not shared. The authors rapidly delivered the OpenSAFELY data analysis platform without previous funding to deliver timely analyses of urgent research questions in the context of the global COVID-19 health emergency: now that the platform is established, the authors are developing a formal process for external users to request access in collaboration with NHS England. Details of this process will be published in the near future on the OpenSAFELY website.
